# The utilization of specialized healthcare services among frail older adults in the Helsinki Birth Cohort Study

**DOI:** 10.1080/07853890.2021.1941232

**Published:** 2021-10-29

**Authors:** Jenni N. Ikonen, Johan G. Eriksson, Minna K. Salonen, Eero Kajantie, Otso Arponen, Markus J. Haapanen

**Affiliations:** a Department of General Practice and Primary Health Care, University of Helsinki, Helsinki, Finland; b Folkhälsan Research Center, Helsinki, Finland; c Department of Obstetrics and Gynecology and Human Potential Translational Research Programme, Yong Loo Lin School of Medicine, National University of Singapore, Singapore; d Agency for Science, Technology and Research, Singapore Institute for Clinical Sciences, Singapore; e Department of Public Health Solutions, Finnish Institute for Health and Welfare, Helsinki, Finland; f Department of Public Health Solutions, THL Finnish Institute for Health and Welfare, Helsinki and Oulu, Finland; g PEDEGO Research Unit, MRC Oulu, Oulu University Hospital, University of Oulu, Oulu, Finland; h Department of Clinical and Molecular Medicine, Norwegian University of Science and Technology, Trondheim, Norway; i Children’s Hospital, Helsinki University Hospital, University of Helsinki, Helsinki, Finland; j Faculty of Medicine and Health Technology, Tampere University, Tampere, Finland; k Department of Radiology, Tampere University Hospital, Tampere, Finland; l Department of Medical Epidemiology and Biostatistics, Karolinska Institute, Stockholm, Sweden

**Keywords:** Frailty, specialized healthcare, hospital services, length of stay, inpatient care, outpatient care

## Abstract

**Background:**

The association between frailty and specialized healthcare utilization is not well studied. We, therefore, examined the utilization of specialized healthcare services among frail Finnish older adults.

**Methods:**

A sub-sample of 1060 participants of the Helsinki Birth Cohort Study were followed prospectively for specialized healthcare utilization from nationwide registers between the years 2013 and 2017. The participants’ frailty status was assessed according to Fried’s criteria at a mean age of 71.0 (2.7 SD) years between the years 2011 and 2013. A negative binomial regression model was used to examine the association between frailty and the total number of visits, emergency visits, outpatient appointments separating the first outpatient appointments and the follow-up appointments, inpatient care including elective and non-elective hospital admissions and the total number of hospital days. We also calculated average length of stay (ALOS) and used the Kruskal–Wallis test to examine the differences between the groups.

**Results:**

After adjusting for covariates, frailty was significantly associated with the number of specialized healthcare visits (IRR 1.50, 95% CI = 1.04–2.15) and all subgroups of visits apart from follow-up outpatient appointments. Frailty was particularly strongly associated with the number of hospital days (IRR 5.24, 95% CI = 2.35–11.7) and notably with emergency visits (IRR = 2.26, 95% CI = 1.45–3.51) and hospital admissions (IRR 2.23, 95% CI = 1.39–3.56). Frail older adults had also higher ALOS compared to non-frail participants (*p* = .009).

**Conclusions:**

Frailty increases the use of most specialized healthcare services. Preventative interventions against frailty are needed to decrease the burden on specialized healthcare systems.

**KEY MESSAGE**
- Frailty is associated with the utilization of most specialized healthcare services, the most expensive part of the healthcare in most high-income countries.
- The association of frailty with inpatient care is particularly strong.
- Preventative interventions against frailty are needed to decrease the burden on specialized healthcare systems.

## Introduction

By the year 2050, the proportion of older adults over 65 years is expected to double to 1.5 billion from the current population of 703 million [1]. Population ageing poses a challenge for healthcare systems and creates a need to understand the patterns of healthcare use among older adults. It is particularly important to recognize the factors that result in frequent healthcare use.

Frailty, referring to the state of exceptional vulnerability to sudden intrinsic factors and exogenous stressors, becomes increasingly prevalent with higher chronological age [2,3]. Frailty is associated with many adverse health outcomes including falls, delirium and premature mortality [2,3] and recognized as a factor leading to increased healthcare utilization [4–13] and costs [13–16]. Comorbidity and disability, known geriatric conditions and risk factors for healthcare utilization among older adults [17–19], are distinct concepts to frailty [20]. However, they overlap with frailty as comorbidities can be considered an etiologic risk factor for frailty and disability a potential outcome of frailty [2]. Therefore, frailty has caught increasing attention during the last decade as it is identified as a potential explaining factor for variety of health issues among older adults [3].

As specialized healthcare offered mainly by hospitals forms a major proportion of health care costs [21], understanding the health service usage behaviour of older adults in specialized health care settings is necessary to plan cost-effective treatments. Currently, length of stay (LOS) is regarded as a key concept of hospital efficiency, and attention has been paid to prefer cheaper outpatient care over costly inpatient care [21]. However, the literature on frailty and its associations with the utilization of specialized healthcare services is scanty. Existing studies have shown divergent results [13,22], focused on the utilization of hospital services as one concept without separating different fields of hospital services [8], been conducted in Asia [23] or taken into consideration only older adults aged over 85 years [4]. Therefore, the purpose of this study is to examine the possible impact of frailty on the utilization of specialized healthcare services including outpatient care, inpatient care, emergency care, hospital days and LOS in a cohort of Finnish older adults.

## Materials and methods

### Study population

**Figure 1. A flowchart of selection of the study population.**
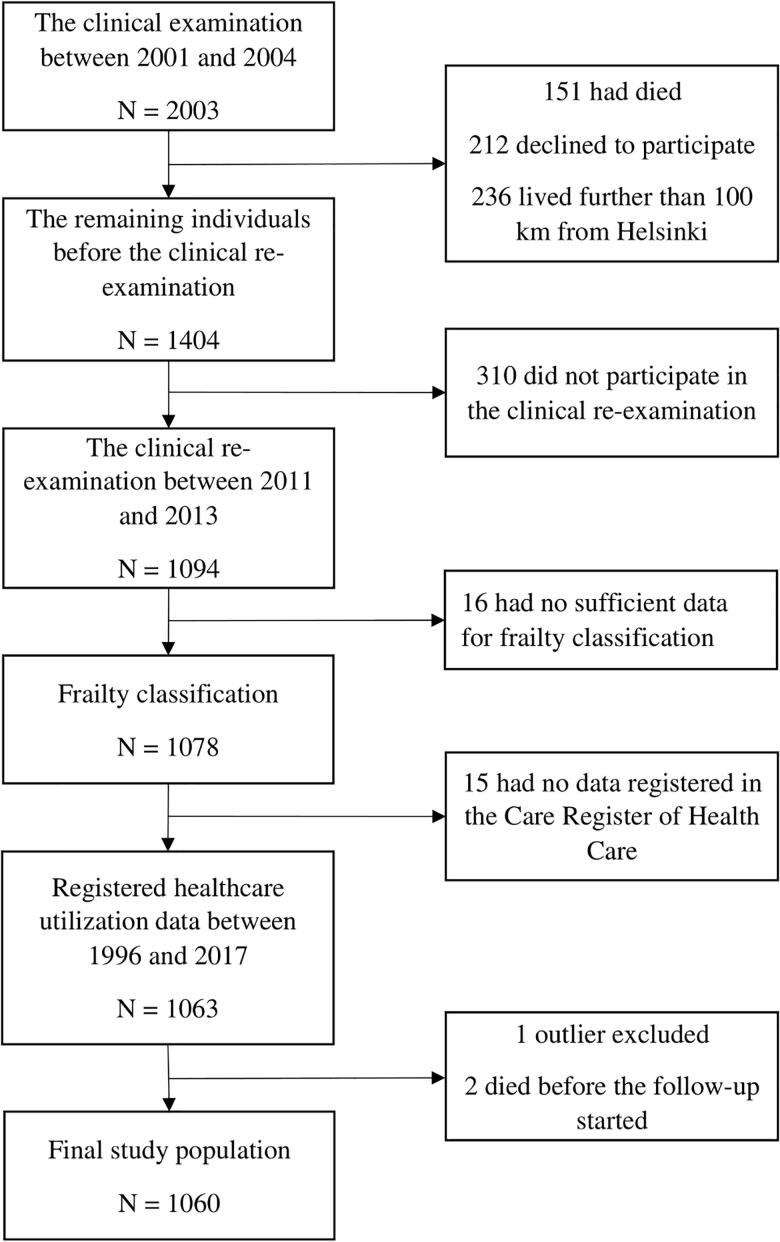


The present study cohort comes from a sub-population of the Helsinki Birth Cohort Study of 8760 individuals born in Helsinki between the years 1934 and 1944 as described previously [24,25]. A random sample of 2003 individuals took part in clinical examinations between the years 2001 and 2004. A clinical re-examination was performed between the years 2011 and 2013. By the clinical re-examination, 151 individuals were deceased, 212 declined to participate in the follow-up study and 236 lived further than 100 km from Helsinki. Of 1404 contacted individuals, 1094 participated in the re-examination. Of re-examined cohort members, 1078 had sufficient data for conducting frailty classification at clinical examinations between the years 2011 and 2013. Of these, 1063 had specialized health care utilization data in a national register called the Care Register of Health Care between the years 1996 and 2017. However, one individual with more than 900 visits (25.9 times the standard deviation (SD)) was identified as an outlier and was thus excluded from the study sample. Moreover, two individuals had died before the data retrieval started for the healthcare utilization in the year 2013. Therefore, the final study population consisted of 1060 individuals as shown in the flowchart in Figure 1. All individuals gave written informed consent before participating in any clinical study procedures. The clinical study was approved by the Coordinating Ethics Committee of The Hospital District of Helsinki and Uusimaa.

### Frailty classification

Frailty was defined according to the five criteria based on Fried’s frailty classification [2]. Weight loss, exhaustion, low physical activity, weakness and slowness were evaluated at the clinical examination between the years 2011 and 2013 as described in detail in previous publications [26–28]. Briefly, a recent weight loss of at least 5 kg, exhaustion on three or more days a week and low physical activity referring to total physical activity of 1 h or less per week were inquired using questionnaires. Weakness was assessed by measuring grip strength and defined as belonging to the weakest quintile of participants according to sex and body mass index (BMI). Slowness was assessed by maximal walking speed stratified by sex and height and defined as belonging to the slowest quintile. Participants were classified frail if they met three or more criteria, pre-frail if one or two criteria and non-frail if they met no criteria.

### Healthcare utilization data

**Figure 2.**
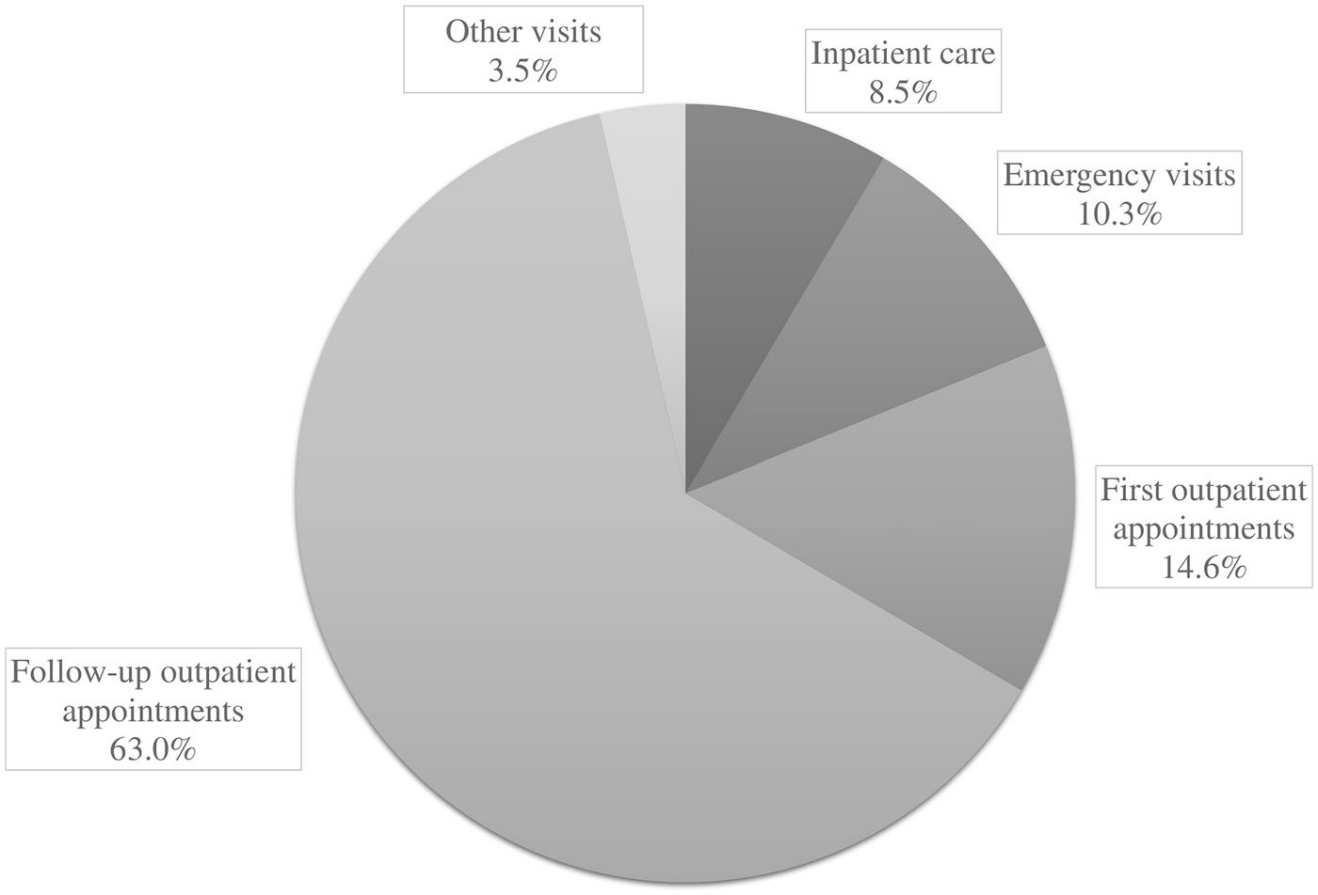
Distribution of the total 15,347 specialized healthcare visits.

The healthcare utilization data were obtained from the Finnish Institute for Health and Welfare which, as a statistical healthcare authority of Finland, maintains the Care Register of Health Care [29]. The register includes all inpatient and outpatient healthcare records of the specialized healthcare in Finland. The specialized healthcare refers to examinations and treatments provided by medical specialists at hospitals and therefore, excludes the medical care of the primary healthcare, for example, visits to general practitioners. To further elucidate, the Finnish healthcare system mainly consists of public healthcare, which includes both primary and specialized healthcare, and to a much smaller extent private healthcare services. The register data consisted of dates for each hospital visit or hospital admission and discharge, ICD-10 diagnosis codes and information on the purpose of the visit, for example, emergency visit, inpatient care. Four biggest subgroups from the total number of visits were retrieved: emergency visits, inpatient care including non-elective and elective hospital admissions and outpatient appointments separating first outpatient appointments and follow-up appointments. The total number of any visit to a specialized health care unit was also calculated and extracted resulting in 15,347 visits. Figure 2 presents the distribution of all visits. Length of stay was calculated by subtracting the hospital admission date from the hospital discharge date and the total sum of hospital days was calculated by adding up the hospital days. Average length of stay (ALOS) was calculated as the mean of all hospital stays. The healthcare records were retrieved from the 1 January 2013 until the 31 December 2017, spanning five years of follow up. The year 2013 was chosen to be the starting point since participants’ frailty status was assessed between the years 2011 and 2013.

### Covariates

Participants’ characteristics were determined during the clinical examinations between the years 2011 and 2013 apart from education which was obtained from Statistics Finland in the year 2000. Height and weight to calculate BMI were measured and results were presented as kilograms divided by the square of height in metres (kg/m^2^). Current smoking status (yes/no) was assessed using a questionnaire. Education was classified into four groups: basic or less or unknown; upper secondary; lower tertiary including polytechnic, vocational and bachelor’s degree; and upper tertiary meaning master’s degree or higher. The Charlson comorbidity index (CCI) [30] was calculated based on the ICD-10 diagnosis codes [31] available between the years 1996 and 2017.

### Statistical analysis

Descriptive statistics of the study population are presented as means and SDs for continuous variables and as proportions for dichotomous or categorical variables. To assess the differences between the groups, the one-way ANOVA test, the Kruskal–Wallis test and Pearson’s chi square test with or without Bonferroni’s correction were used when appropriate. As data were overdispersed, we used a negative binomial model to model the number of visits of each subdomain in the study population. Due to natural deaths, the follow-up time varied among participants and, therefore, individually calculated log-transformed time of follow-up was set as an offset variable to the model. To test the assumption of overdispersion and preference of the negative binomial model over the Poisson model, the Lagrange multiplier test was used. Model 1 is the crude model. Model 2 was additionally adjusted with age and sex and model 3 further with education. Model 4 includes the previous covariates and additionally BMI, smoking status and CCI. The results are presented as incidence rate ratios (IRRs). The variance inflation factor test was used to assess possible multicollinearity between the predicting variables. Significance was set at *p* ˂ .05. SPSS 24.0 for Windows (Version 24.0, 1989–2020, SPSS Inc., Armonk, NY) was used for statistical analyses.

## Results

### General characteristics

The characteristics of 1060 study participants are presented in Table 1. Frail participants scored higher in CCI (*p*<.001) and had higher BMI (*p* = .002) compared to non-frail ones. Most frail individuals had basic education or less (*p*<.001). Incident mortality was low to moderate (*n* = 62, 5.9%) during the five-year follow-up.

**Table 1.** Characteristics of the study population by frailty phenotype.

	All		Non-frail		Pre-frail		Frail		
	*n*	Mean (SD)	*n*	Mean (SD)	*n*	Mean (SD)	*n*	Mean (SD)	*p* Value
Characteristics									
Age (years)	1060	71.0 (2.7)	597	70.6 (2.4)	425	71.4 (3.0)	38	71.3 (2.3)	.002
Height (cm)	1049	168.4 (9.1)	592	168.9 (9.0)	420	168.0 (9.2)	37	165.3 (8.9)	.027
Weight (kg)	1049	76.9 (14.3)	592	75.9 (13.2)	420	78.2 (15.4)	37	76.9 (17.2)	.16
BMI (kg/m^2^)	1049	27.1	592	26.6 (4.0)	420	27.7 (4.9)	37	28.2 (6.4)	.002
Women, %	594	56.0	332	55.6	236	55.5	26	68.4	.29
Smoker, %	120	11.3	50	8.4	65	15.5	5	13.5	.003
CCI	1060	3.53 (1.4)	597	3.40 (1.4)	425	3.65 (1.3)	38	4.24 (1.7)	˂.001
Education									˂0.001
Basic or less or unknown, %	341	32.2	161	27.0	163	38.4	17	44.7	
Upper secondary, %	268	25.3	143	24	114	26.8	11	28.9	
Lower tertiary, %	297	28.0	192	32.2	98	23.1	7	18.4	
Upper tertiary, %	154	14.5	101	16.9	50	11.8	3	7.9	
Healthcare utilization during the follow-up									
Visits/year	1060	3.40 (6.3)	597	2.94 (5.7)	425	3.75 (6.8)	38	6.62 (9.3)	˂.001
Hospital admissions/year	1060	0.33 (1.0)	597	0.23 (0.54)	425	0.42 (1.4)	38	0.93 (1.5)	˂.001
Emergency visits/year	1060	0.41 (1.8)	597	0.36 (2.2)	425	0.41 (0.93)	38	1.08 (2.0)	˂.001
First outpatient appointments/year	1060	0.46 (0.55)	597	0.39 (0.39)	425	0.51 (0.63)	38	0.92 (1.1)	˂.001
Follow-up outpatient appointments/year	1060	2.12 (4.8)	597	1.90 (4.5)	425	2.30 (5.2)	38	3.56 (5.8)	˂.001
Hospital days/ year	1060	2.61 (15.8)	597	1.32 (5.5)	425	3.87 (23.3)	38	8.68 (19.3)	˂.001
ALOS (days)	454	6.21 (11.0)	227	5.57 (10.6)	203	6.40 (10.8)	24	8.46 (13.7)	.009

SD: standard deviation; BMI: body mass index; ALOS: average length of stay.

### Healthcare utilization

**Figure 3.**
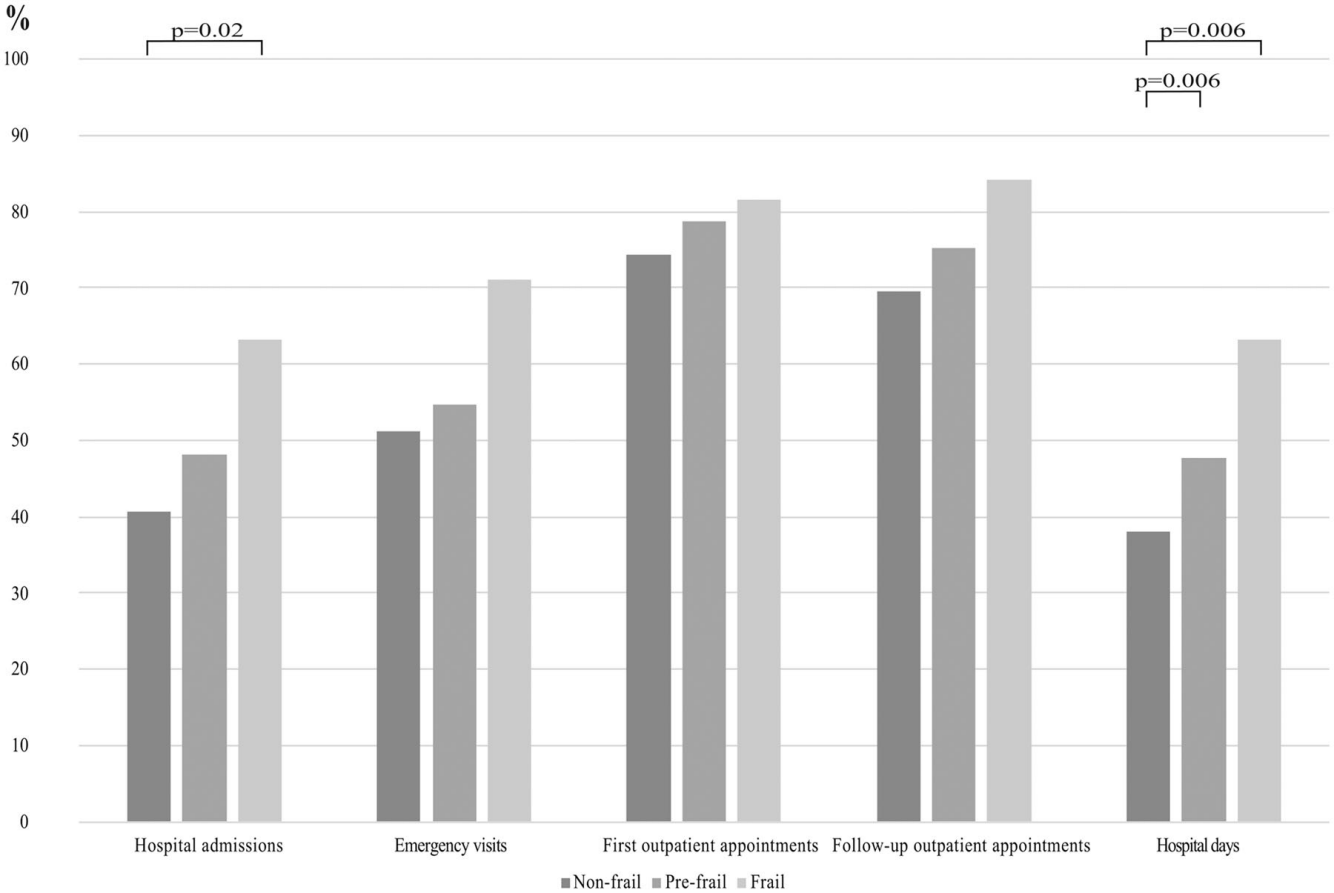
Proportion of the utilization of healthcare services by frailty phenotype. Significance shown for the pairwise comparisons.

Figure 3 presents the proportion of utilization of healthcare services by those exhibiting frailty phenotype. Among those with first outpatient appointments, follow-up outpatient appointments were seen in 93.5% of non-frail, 95.5% of pre-frail and 100% of frail, respectively. Frail older adults were characterized by significantly higher rates of health care utilization during the follow-up than the non-frail participants; they had more visits, hospital admissions, emergency visits, first outpatient appointments, follow-up outpatient appointments and hospital days adjusted with individual time of follow-up (years) (*p*<.001) and longer ALOS (*p* = .009) (Table 1).

### Total healthcare utilization and frailty classification

Table 2 shows that frailty was associated with the utilization of almost all types of specialized healthcare services. Frailty was significantly associated with the total number of visits (IRR 1.92, 95% CI 1.27–2.90) and the association remained statistically significant after further adjustments. However, although remaining significant, the results attenuated slightly after further adjusting for CCI, BMI and smoking (IRR 1.50, 95% CI 1.04–2.15).

**Table 2.** Incidence rate ratios (IRRs) of the negative binomial regression models for the utilization of specialized healthcare services among frail Finnish older adults between the years 2013 and 2017.

	Model 1^a^	Model 2^b^	Model 3^c^	Model 4^d^
	IRR (95% CI)	IRR (95% CI)	IRR (95% CI)	IRR (95% CI)
Total number of visits				
Non-frail	Ref.	Ref.	Ref.	Ref.
Pre-frail	1.32 (1.13, 1.55)**	1.31 (1.12, 1.53)**	1.28 (1.09, 1.50)**	1.16 (1.00, 1.33)*
Frail	1.92 (1.27, 2.90)**	2.01 (1.34, 3.03)**	2.02 (1.34, 3.04)**	1.50 (1.04, 2.15)*
Hospital admissions				
Non-frail	Ref.	Ref.	Ref.	Ref.
Pre-frail	1.56 (1.25, 1.95)***	1.57 (1.25, 1.94)***	1.46 (1.17, 1.83)**	1.26 (1.02, 1.55)*
Frail	3.22 (1.86, 5.56)***	3.34 (1.93, 5.75)***	3.16 (1.83, 5.46)***	2.23 (1.39, 3.56)**
Emergency visits				
Non-frail	Ref.	Ref.	Ref.	Ref.
Pre-frail	1.36 (1.12, 1.64)**	1.34 (1.10, 1.62)**	1.29 (1.06, 1.56)*	1.15 (0.95, 1.39)
Frail	3.02 (1.88, 4.84)***	3.01 (1.88, 4.82)***	2.94 (1.83, 4.72)***	2.26 (1.45, 3.51)***
First outpatient appointments				
Non-frail	Ref.	Ref.	Ref.	Ref.
Pre-frail	1.25 (1.11, 1.42)***	1.23 (1.09, 1.39)**	1.22 (1.08, 1.38)**	1.19 (1.06, 1.35)**
Frail	1.91 (1.42, 2.56)***	1.89 (1.41, 2.53)***	1.88 (1.40, 2.52)***	1.63 (1.23, 2.16)**
Follow-up outpatient appointments				
Non-frail	Ref.	Ref.	Ref.	Ref.
Pre-frail	1.29 (1.05, 1.59)*	1.28 (1.05, 1.57)*	1.25 (1.02, 1.53)*	1.15 (0.96, 1.39)
Frail	1.60 (0.93, 2.74)	1.74 (1.02, 2.98)*	1.76 (1.02, 3.02)*	1.29 (0.80, 2.08)
Hospital days				
Non-frail	Ref.	Ref.	Ref.	Ref.
Pre-frail	2.00 (1.43, 2.80)***	1.99 (1.41, 2.79)***	1.90 (1.35, 2.68)***	1.71 (1.24, 2.36)**
Frail	5.22 (2.16, 12.6)***	5.68 (2.34, 13.8)***	5.74 (2.36, 14.0)***	5.24 (2.35, 11.7)***

CI: confidence interval.

*p<.05.

**p<.01.

***p<.001.

^a^ Crude model, adjusted for frailty, *n* = 1060.

^b^ Adjusted for frailty, age and sex, *n* = 1060.

^c^ Adjusted for frailty, age, sex and education, *n* = 1060.

^d^ Adjusted for frailty, age, sex, education, BMI, smoking and CCI, *n* = 1048.

### Categories of healthcare utilization and frailty classification

#### Hospital admissions and number of hospital days

The impact of frailty was strongly seen in hospital admissions (IRR 3.22, 95% CI 1.86–5.56) and in number of hospital days (IRR 5.22, 95% CI 2.16–12.6). Adjustment with CCI, BMI and smoking weakened the association between frailty and hospital admissions slightly (IRR 2.23, 95% CI 1.39–3.56) but the results remained significant. Further adjustments had minor impact on the association between frailty and the hospital days (in fully adjusted model IRR 5.24, 95% CI 2.35–11.7). The associations were weaker but parallel among pre-frail individuals (Table 2).

#### Number of emergency visits, first outpatient appointments and follow-up outpatient appointments

Frailty was notably associated with the use of emergency visits (IRR 3.02, 95% CI 1.88–4.84) compared with pre-frail and non-frail participants (Table 2). The association slightly attenuated after adjustments with CCI, BMI and smoking (IRR 2.26, 95% CI 1.45–3.51) but the association remained significant. Frailty was also associated with the number of first outpatient appointments (IRR 1.91, 95% CI 1.42–2.56). In contrast, there was no association between frailty and follow-up outpatient appointments (Table 2). Although remaining significant, after further adjusting for CCI, BMI and smoking, the association between frailty and first outpatient appointments attenuated slightly (IRR 1.63, 95% CI 1.23–2.16). Compared to frailty, the associations between pre-frailty and emergency visits and first outpatient appointments were weaker but parallel (Table 2). Moreover, there was no association between pre-frailty and follow-up outpatient appointments (Table 2).

## Discussion

This prospective cohort study combining clinical information and national register data shows that frailty was associated with the utilization of almost all types of specialized healthcare services in Finland. The association was particularly strong with the total number of hospital days but was also notably seen in the other investigated subgroups including hospital admissions, emergency visits and first outpatient appointments. As a new finding we present that frailty was associated with first outpatient appointments while there was no association between frailty and follow-up outpatient appointments. We also report that frail individuals had longer ALOS compared to non-frail individuals.

Apart from one study based on a national database [4], previous studies about the association of frailty with the utilization of healthcare services are based on questionnaires [7,12] or surveys [5,8–11] or conducted with selected patient populations [6,22] or hospitals [13,23] creating a need for further nationwide register studies. Additionally, no studies have assessed frailty and its possible associations with healthcare use in countries, like Finland, where the quality of healthcare is regarded high and the healthcare system is heavily subsidized. Moreover, Finland has one of the oldest populations in Europe, one of five being 65 years or older, and the share is predicted to increase to 29% by the year 2060 [32].

In our study, the prevalence of frailty was 3.6% among community-dwelling older adults. This is less than the weighted average estimate of 11.0% for frailty in a systematic review which examined studies about community-dwelling older adults in high income countries [33]. However, the same review reported a range from 4.0% to 22.7% in the studies applying Fried’s frailty scale to assess frailty [33]. Therefore, the prevalence is similar to the lowest value of the range reported in the review. It must be pointed out, however, that the participation to the clinical re-examinations was voluntary, and it might have negatively affected the participation rate among those with poor health.

Frailty was a strong predictor of inpatient care including hospital admissions and especially the number of in-hospital days. Supporting our findings, several previous studies have found frailty to be associated with the number of hospital admissions [5–12,23]. Furthermore, the association has been established in a large meta-analysis of 13 countries [34]. However, little literature exists regarding the association of frailty with elective and non-elective admissions. Keeble et al. investigated the number elective and non-elective admissions among older adults and found that frail individuals tended to have more non-elective admissions whereas non-frail individuals had more elective admissions [4]. Notably, our records of hospital admissions included both acute and elective admissions and, therefore, our results are not directly comparable. Nevertheless, the vulnerability of frail individuals for acute stress factors might predispose them to need the services offered by wards in both acute and elective situations.

As in our study, some studies have also found the association of frailty with the total number of hospital days within a study follow-up [4,6,7]. Prolonged LOS among frail individuals, in turn, is well-documented in the literature [35–39] and confirmed in a recent meta-analysis [40]. We also found that ALOS was significantly longer among frail than non-frail individuals. Since frail individuals are characterized by poor homeostatic responses following stress [2,3], they might be prone to develop secondary health outcomes after hospital admission leading to an increased LOS and, in the long run, increased total number of hospital days. Further, LOS is an important indicator of the hospital efficiency, and inpatient care recognized as the most expensive part of the hospital services [21]. Therefore, geriatric interventions for preventing frailty might lead to economical savings by reducing the number of hospital admissions, LOS and total number of hospital days.

Frailty was also associated with emergency visits. The literature regarding frailty and emergency visits, however, is controversial. While some studies have reported increased utilization of emergency services among frail individuals [6,8,11,41,42], others have not [7,43]. Interestingly, in an Italian study, the association was seen until the model was adjusted with Basic Activities of Daily Living (BADL) representing disability [43], and the association also disappeared in an Irish study after adjusting with several covariates including Instrumental of Activities of Daily Living (IADL) representing disability [7]. Disability increases the use of emergency services among older adults [44–46], and therefore, disability might have an impact on the number of emergency visits. Our study population, however, consisted of older adults who lived in their own homes and their willingness to participate was based on voluntariness. Thus, it is unlikely that they would have scored high in BADL or IADL, and the differences between different studies may be due to other confounding factors, for instance, differences in used frailty classifications or demographical factors.

Frailty was associated with increased odds of first outpatient appointments, but we observed no association between frailty and follow-up outpatient appointments. Several previous studies have found frailty to be associated with outpatient appointments [5,7,8,11,23] whereas Keeble et al. found elective visits to be more common among non-frail in a 7-year follow-up [4] and García-Nogueras et al., in turn, did not find any association [13]. Previous studies, however, have not separated outpatient care into first and follow-up outpatient appointments. Our result suggests that frail older adults get more referrals to specialized healthcare to visit a specialist than non-frail older adults. On the other hand, in some cases, further specialized healthcare services might not be considered necessary leading to the same rate of utilization of follow-up outpatient appointments among older adults. Possible influential factors to this pattern might be either straightforward treatments or comorbidities that may raise concern, but which may be more suitable for general practitioner follow-ups considering the overall health of a frail patient.

Finally, the literature on frailty with the focus solely on the total utilization of specialized healthcare or hospital services is scarce. In contrast to our finding, a regional register-based study conducted in Lazio region in Italy found that the highest rate of the utilization of hospital services was among pre-frail individuals [22]. However, authors declared that their frailty questionnaire might not be the best predictor of the utilization of hospital services and pointed out that Italy has one of the lowest hospital inpatient admission rates in Europe. Supporting our study, frailty was associated with the utilization of hospital services in a population-based cohort study in the UK [4] and in an Australian cross-sectional surveillance study [8].

Although frailty was associated with almost all types of specialized healthcare services, we found that most associations attenuated slightly after adjusting for BMI, CCI and smoking status. Higher BMI increases the utilization of specialized healthcare services among US population aged over 65 years [47], and smokers utilize more healthcare services than non-smokers [48–50]. Comorbidity, in turn, increases the healthcare utilization among older adults [17,18]. Therefore, the attenuated response could be due to the individual effect of each of these covariates. Alternatively, a part of the increased utilization of healthcare services associated with frailty could also be attributed to common underlying causes including BMI, comorbidity and smoking [3]. Nevertheless, although healthcare utilization increases with age [51], and various other factors including demographical and social factors drive older adults to seek healthcare services [52,53], interventions on potentially modifiable factors associated with healthcare utilization could delay or prevent clinical consequences. This is particularly important in countries, like Finland, where the expenses of specialized healthcare and pharmaceuticals have led an increase in GDP spent on health during the past decade, and the growth is predicted to increase due to population ageing [54].

Overall, frailty was associated with the use of almost all types of specialized healthcare services. It highlights the importance of adapting specialized healthcare services according to the needs of the rapidly increasing number of frail older adults. On the other hand, despite the expertise in specialized healthcare, inpatient care among frail older adults is associated with adverse health outcomes including mortality and functional decline at discharge [40]. Moreover, frail older adults have higher number of hospital admissions and longer LOS than non-frail older adults leading to higher healthcare expenses and decreased number of available hospital beds. Our study also suggests that frail older adults get more referrals to a specialist. Some of the follow-up outpatient appointments, however, might not be considered necessary referring possibly to straightforward treatments or comorbidities that are, after all, more suitable for general practitioner follow-ups. Nevertheless, these findings emphasize the role of frailty as a potential target for preventative interventions to maintain cost-efficiency and sustainability in specialized healthcare. Physical, nutritional and cognitive interventions in particular have shown promising effects on reversing frailty [55,56] and may play an important role in healthy ageing in the future.

The strengths of our study are the use of national register-based data, which makes the data reliable in comparison with questionnaires, and the study population which is part of the phenotypically richest and oldest births cohorts in the world [24]. Some limitations must also be noted. First, the participation in the clinical re-examinations was voluntary and those who declined might have done it for health reasons. Therefore, our study sample may underestimate the real prevalence of frailty and the participants may represent the healthiest sub-sample of the birth cohort. It could also lead to underestimation of the use of specialized healthcare services as the frail older adults had the highest rate of utilization. Second, our study examines the utilization of specialized health care services of older adults in a country where the health care standards are considered globally high. The results could offer valuable information to Nordic countries where the proportion of older adults is high and, therefore, the demography similar. Instead, the results might not be as easily generalizable to other countries. Finally, the frailty classification might have changed during the follow-up leading to slightly mispresented results.

In conclusion, in our prospective cohort study, we confirmed that frailty was associated with the utilization of almost all types of specialized healthcare services among older adults in Finland. We also offered new information on the association between frailty and outpatient care. Further research, however, is needed to establish our findings especially with the associations of frailty with the total use of specialized healthcare services and utilization of outpatient care. Preventative health interventions against frailty are needed to face the challenges of ageing populations in specialized healthcare.

## Data Availability

Inquiries regarding the datasets used and/or analysed during the current study can be directed to the principal investigator (Johan G. Eriksson) of the Helsinki Birth Cohort Study.
